# Increasing the Medium Osmolarity Reduces the Inflammatory Status of Human OA Chondrocytes and Increases Their Responsiveness to GDF-5

**DOI:** 10.3390/ijms21020531

**Published:** 2020-01-14

**Authors:** Tanja Mang, Sven Lindemann, Anne Gigout

**Affiliations:** 1 Osteoarthritis Research, Merck KGaA, 64293 Darmstadt, Germany; tanja.mang@web.de (T.M.); sven.lindemann@merckgroup.com (S.L.); 2 Institute for Organic Chemistry and Biochemistry, Technische Universität, 64287 Darmstadt, Germany

**Keywords:** osmolarity, osteoarthritis, chondrocytes, growth and differentiation factor-5 (GDF-5)

## Abstract

The environment surrounding chondrocytes changes drastically in osteoarthritis (OA). For instance, the osmolarity in cartilage (ranging from 350 to 460 mOsm in healthy tissue) decreases during the progression of OA, reaching 270 mOsm. The objective of this study was to evaluate how osmolarity influences human OA chondrocytes. For this purpose, the osmolarity of the culture medium (340 mOsm) was increased to 380, 420 or 460 mOsm and its effect on the phenotype, matrix production, protease expression, cytokine release and growth and differentiation factor-5 (GDF-5) receptor expression in human OA chondrocytes was evaluated in a monolayer. Afterwards, the same parameters, as well as the responsiveness to GDF-5, were evaluated in 3D culture at 340 and 380 mOsm. Our results revealed that increasing the medium osmolarity increased matrix production but also reduced cytokine release, type I collagen and protease expression. It was also demonstrated that at 380 mOsm, the response to GDF-5 in 3D culture was more robust than at 340 mOsm. For the first time, it was established that a decreased osmolarity plays a role in sustaining inflammation and catabolic activities in OA chondrocytes and decreases their responsiveness to GDF-5. This indicates that osmolarity is a critical aspect of OA pathobiology.

## 1. Introduction

Osteoarthritis (OA) is the most common chronic joint disease, where the symptoms include pain and loss of mobility of the affected joint, resulting in a significantly reduced quality of life [1]. OA is characterized by the degradation of articular cartilage, which is mediated by an elevated catabolism and an inappropriate repair response of the chondrocytes.

In contrast to healthy chondrocytes, OA chondrocytes show a large heterogeneity of phenotypes displaying hypertrophic, dedifferentiated, inflammatory and/or catabolic features [2]. In addition, human OA chondrocytes have been shown to be less responsive to anabolic stimulation. Indeed, OA chondrocytes showed a lower response to IGF1 [3] and TGF*β* [4], or a discordant response to GDF-5 [5]. These changes in the chondrocyte phenotype during OA occur concurrently with drastic modifications of the cell environment. OA chondrocytes are submitted to abnormal loading, inflammatory conditions and an altered extracellular matrix (ECM) [2]. For instance, the proteoglycan loss in OA tissue results in a decreased fixed charge density and decreased osmolarity [6], reaching 270 mOsm [7,8] in OA tissue, and ranging from 350 to 480 mOsm in healthy cartilage [9]. Thus, the osmolarity within the cartilage is directly connected to the OA stage. Yet, chondrocytes are known to respond favorably to osmolarities that correspond to healthy cartilage. Indeed, it was shown that at osmolarities of 350–400 mOsm, the ECM molecule production was increased, and type I collagen expression decreased in comparison to lower osmolarities of 250 to 280 mOsm [10,11,12,13].

In the present study, we hypothesized that an osmolarity corresponding to healthy cartilage could attenuate the OA characteristics of chondrocytes isolated from late OA cartilage and possibly improve their responsiveness to growth factors. For this purpose, GDF-5 was used. GDF-5 belongs to the bone morphogenetic protein family (BMPs) and signal through the BMP receptor BMPR2 together with BMPR1a or BMPR1b [14]. GDF-5 was shown to protect against OA in a rat medial meniscal tear (MTT) model [15] and to have anabolic activities in healthy human chondrocytes [16,17,18,19]. However, another study showed that OA chondrocytes from different donors respond differently to GDF-5 leading to an overall discordant response [5]. 

To verify our hypothesis, we first investigated the effect of increased osmolarities (340 versus 380, 420 and 460 mOsm) on matrix production, the phenotype and the inflammatory status of human OA chondrocytes in a monolayer and confirmed these results in 3D culture at 340 versus 380 mOsm. Afterwards, the responsiveness of human OA chondrocytes to GDF-5 at 340 or 380 mOsm was compared. Finally, it was also evaluated whether the culture system itself (2D versus 3D) and the inflammatory status of the cells has an impact on BMPR expression and therefore the responsiveness of the cells to GDF-5. For each experiment, between 3 and 8 donors were used and for each donor three to six cell culture replicates were performed for each measured parameter (called technical replicates).

## 2. Results

### 2.1. Increasing the Medium Osmolarity from 340 to 460 mOsm Stimulates Matrix Production and Reduces Type I Collagen Expression in Human OA Chondrocytes in Monolayer Culture

**Figure 1:**
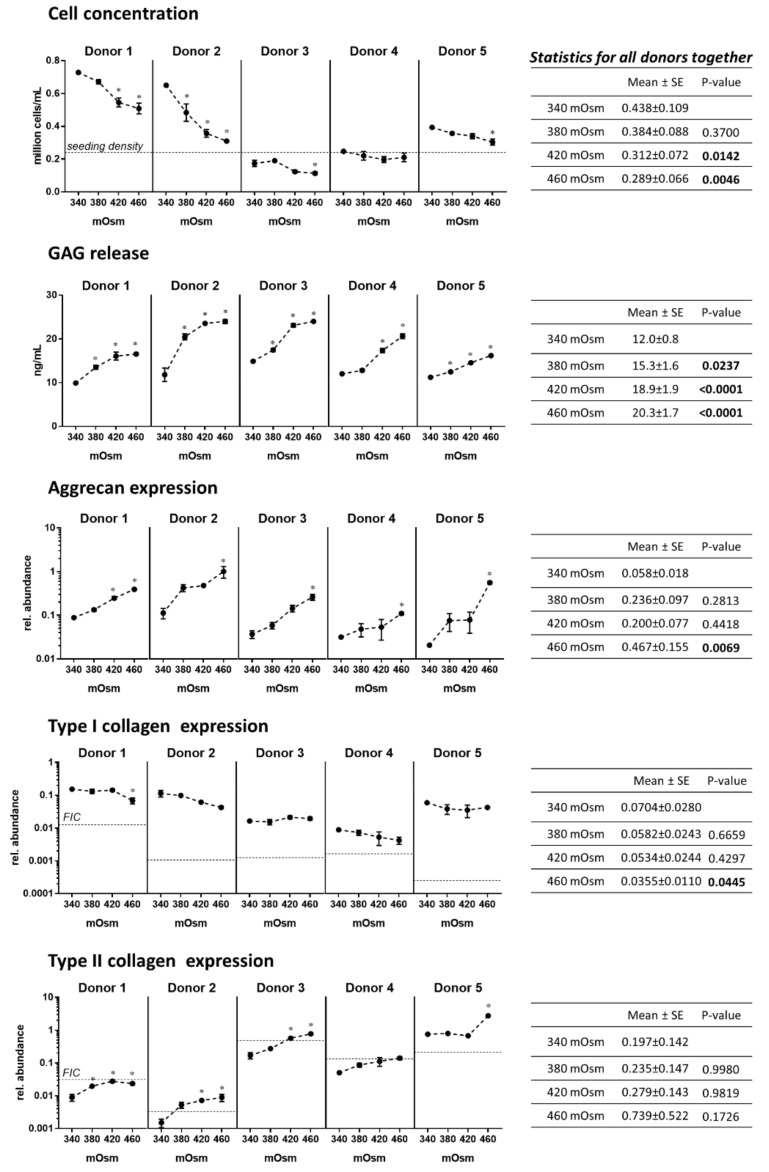
Human OA chondrocytes from five donors were cultured for six days in a monolayer at 340, 380, 420 and 480 mOsm. The cell concentration and the GAG release in the medium were measured (see Materials and Methods). Aggrecan, and type II and I collagen gene expression were evaluated by RT-qPCR. Data on the graphs represent the mean +/- standard error of the mean of technical replicates (*n* = 3–6). For the cell concentration, the seeding density is shown with a dotted line as well as the level of gene expression in freshly isolated chondrocytes (FIC) for type I and II collagen. Statistical analysis was performed for each donor separately (* means significantly different from 340 mOsm with *p* < 0.05) and for all donors together with the means, standard errors of the mean (SE) and *p* values for the comparison to 340 mOsm being tabulated on the right (*p* values in bold when *p* ≤ 0.05).

Results presented in Figure 1 show that increasing the medium osmolarity from 340 to 380, and 420 or 460 mOsm, significantly increased glycosaminoglycan (GAG) release (measured in the medium with the dimethylmethylene blue assay, see paragraph 4.7) by human OA chondrocytes in monolayer culture. This effect was robust and could be observed in all five tested donors. In addition, in all donors, aggrecan and type II collagen expression increased with increasing osmolarity. The statistical analysis including all donors showed that GAG release was significantly increased at all osmolarities compared to 340 mOsm (*p* = 0.0237 at 380 mOsm and *p* < 0.0001 at 420 and 460 mOsm) and aggrecan expression was significantly increased at 460 mOsm (*p* < 0.0069). Overall, it can be concluded that higher osmolarities positively influence the synthesis of ECM molecules.

Increasing osmolarity also reduced type I collagen expression in two of the five donors, indicating a beneficial effect of higher osmolarities on the chondrocyte phenotype (Figure 1). When taking all donors into account, a significant difference was observed at 340 versus 460 mOsm (*p* = 0.0445). To better appreciate how osmolarity and monolayer culture influence the chondrocyte phenotype, the expression levels of type I and II collagen in freshly isolated chondrocytes (FIC) is shown as a dotted line in Figure 1. When comparing the expression levels in FIC and in chondrocytes cultured one week in unchanged culture medium (at 340 mOsm), type I collagen increased (all five donors) and type II collagen decreased (donors 1–4). This corresponds to the already well described chondrocyte dedifferentiation in a monolayer. Increasing the osmolarity partially reverses this effect with type I and II collagen expression levels being closer to these of FIC expression levels at higher osmolarities than at 340 mOsm.

Finally, six days of monolayer culture at osmolarities of 420 and 460 mOsm resulted in a significantly lower cell concentration (*p* = 0.0142 and *p* = 0.0046, respectively) in comparison to 340 mOsm (Figure 1). For donor 1, 2 and 5, cell proliferation occurred at all osmolarities as the final cell concentration was higher than the seeding density. The lower final cell density at higher osmolarity can be explained by a reduced proliferation combined with a slightly lower cell viability (supplementary Figure S1) compared to 340 mOsm. For donor 3 and 4, there was no increase in the cell concentration compared to the seeding density, indicating that these cells were not proliferating. No impact of osmolarity on the cell viability could be observed for these two donors (Supplementary Figure S1). 

### 2.2. Increasing the Medium Osmolarity from 340 to 460 mOsm Attenuates the Protease Expression and Cytokine Release in Human OA Chondrocytes in Monolayer Culture

Human OA chondrocytes were found to express ADAMTS5, MMP13 and produce IL1*β*, TNF*α* and IL6 in accordance with their OA phenotype. Interestingly the most abundant cytokine was IL6 followed by TNF*α* and then IL1*β*.

**Figure 2:**
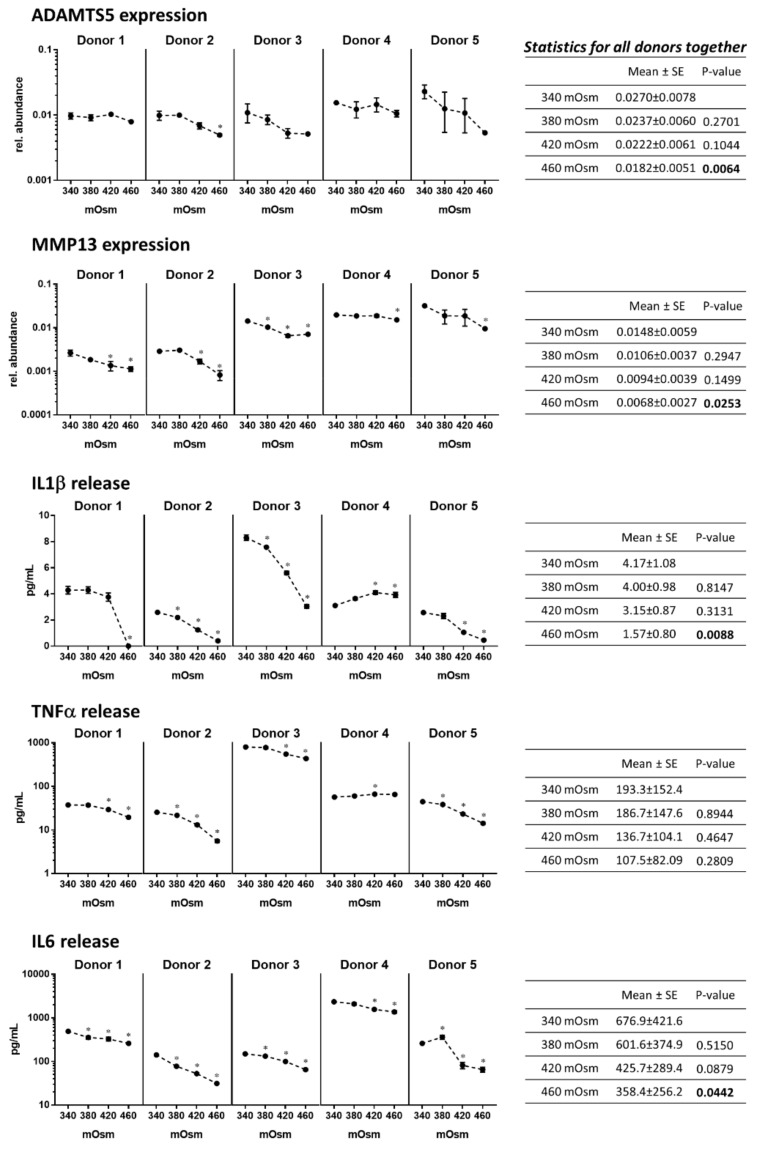
Human OA chondrocytes from five donors were cultured for six days in a monolayer at 340, 380, 420 and 480 mOsm. The gene expression of ADAMTS5 and MMP13 was evaluated by RT-qPCR and the release of IL1*β*, IL6 and TNF*α* was evaluated with a cytokine multiplex assay (see Materials and Methods). Data on the graphs represent the mean -standard error of the mean of technical replicates (*n* = 3–6). Statistical analysis was performed for each donor separately (* means significantly different from 340 mOsm with *p* < 0.05) and for all donors together with the means, standard errors of the mean (SE) and *p* values for the comparison to 340 mOsm being tabulated on the right (*p* values in bold when *p* ≤ 0.05).

Increasing osmolarity from 340 to 460 mOsm significantly decreased ADAMTS5 (*p* = 0.0064 at 460 mOsm) and MMP13 (*p* = 0.0253 at 460 mOsm) expression in human OA chondrocytes (Figure 2). Increasing the medium osmolarity also significantly decreased TNF*α*, IL1*β* and IL6 release (Figure 2). This effect was robust and could be observed in all five tested donors. The statistical analysis including all donors showed that IL1*β* and IL6 were significantly decreased at 460 mOsm (*p* = 0.0088 and *p* = 0.0442, respectively). These results indicate that increasing osmolarity from 340 to 460 mOsm reduces the ‘OA’ phenotype of the cells. 

To verify that the effects of osmolarity on matrix production, the cell phenotype, the protease expression and cytokine release were not NaCl effects but real osmolarity effects, the same experiment was performed with sucrose with two additional donors. Most of the effects observed with NaCl could be reproduced with sucrose (Supplementary Figure S2).

### 2.3. Increasing the Osmolarity from 340 to 380 mOsm in 3D Culture Stimulates Matrix Production and Reduces Type I Collagen Expression in Human OA Chondrocytes

**Figure 3:**
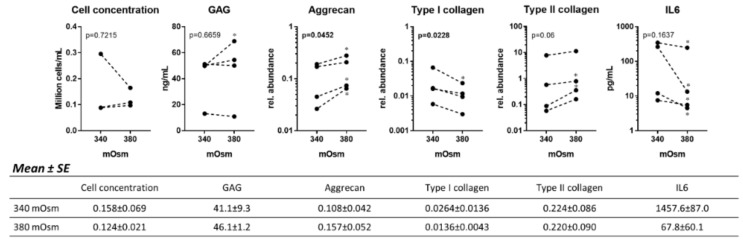
Human OA chondrocytes were cultured for three weeks in alginate at 340 or 380 mOsm. The alginate beads were dissolved, and the cell and GAG content measured in the alginate. Cells were also used to evaluate gene expression of aggrecan, type II and I collagen by qRT-PCR. IL6 was measured in the medium with a cytokine multiplex assay (see Materials and Methods). Data on the graphs represent the mean of technical replicates (*n* = 3–6). Statistical analysis was performed for each donor separately (* means significantly different from 340 mOsm with *p* < 0.05) and for all donors together with the *p* values being shown on the graphs (*p* values in bold when *p* ≤ 0.05). The means and standard errors of the mean (SE) for all donors together are tabulated below the graphs.

The effect of an increased osmolarity was then investigated in 3D alginate culture at 340 versus 380 mOsm (Figure 3). 

Increasing the osmolarity to 380 mOsm had no effect on the cell count or GAG release but significantly increased the expression of aggrecan in human OA chondrocytes (*p* = 0.0452). This effect was robust and observable in all tested donors. In addition, in all four donors, type II collagen expression was increased at 380 compared to 340 mOsm (significant for two donors, *p* = 0.06 for all donors together) and type I collagen expression was significantly decreased (*p* = 0.0228 for all donors together).

Additionally, it could be demonstrated that human OA chondrocytes in 3D culture also express ADAMTS5, MMP13 and produce IL1*β*, TNF and IL6 (not shown). Similar to the monolayer culture, IL6 was the most abundant cytokine followed by TNFα and finally IL1*β*. Interestingly, the cytokine levels were lower in 3D culture than in monolayer culture (not shown). This indicates that the cells lose their inflammatory status with time (in the 3D cultures, cells were cultured for a total period of four weeks but only six days for the monolayer culture). On the other hand, the levels of expression of ADAMTS5 and MMP13 were similar in 3D and monolayer cultures.

Increasing the osmolarity from 340 to 380 mOsm had no significant effect on MMP13 and ADAMTS5 expression or on TNFα or IL1*β* release (not shown). However, the IL6 production was reproducibly decreased in all four donors at 380 mOsm (significant for all donors).

### 2.4. Increasing the Medium Osmolarity from 340 to 380 mOsm in 3D Culture Improves the Responsiveness of Human OA Chondrocytes to GDF-5

**Figure 4:**
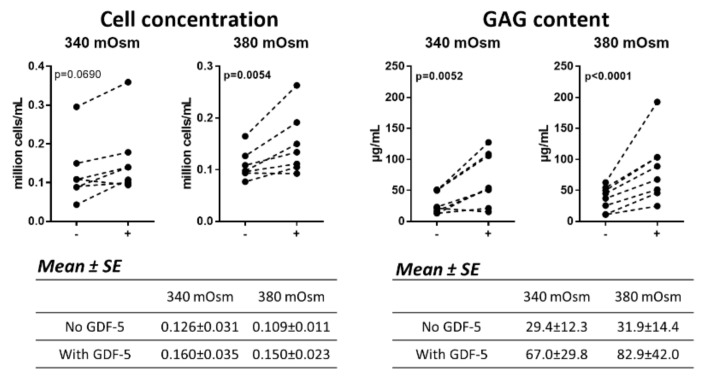
Human OA chondrocytes were cultured for three weeks in alginate at 340 or 380 mOsm and were left untreated (-) or were treated for two weeks with GDF-5 at 300 ng/mL (+). The alginate beads were then dissolved, and the cell and GAG content measured in the alginate. Data on the graphs represent the mean of technical replicates (*n* = 3–6). Statistical analysis was performed on all donors together (*n* = 7–8 donors). *p* values are shown on the graphs and are bold when *p* ≤ 0.05. The means and standard errors of the mean (SE) for all donors together are tabulated below the graphs.

To further evaluate the effect of osmolarity in 3D culture, whether the responsiveness of human OA chondrocytes to GDF-5 was different at 380 mOsm compared to 340 mOsm was investigated (Figure 4).

The addition of GDF-5 significantly enhanced the cell concentration at 380 mOsm (*p* = 0.0054) but not at 340 mOsm (*p* = 0.0690) compared to untreated cells. In addition, the concentration of GAG was enhanced by GDF-5 at both 340 mOsm (*p* = 0.0052) and 380 mOsm (*p* < 0.0001). For both the cell and the GAG concentration, the *p* values for all donors together were lower at 380 mOsm, indicating that the response was more pronounced at this osmolarity. Indeed, in two and four of the donors, the effect of GDF-5 on the cell or GAG concentration, respectively, was stronger at 380 mOsm compared to 340 mOsm (not shown).

### 2.5. The Culture System and the Osmolarity Influence the Expression of BMPRs in Human OA Chondroyctes

**Figure 5:**
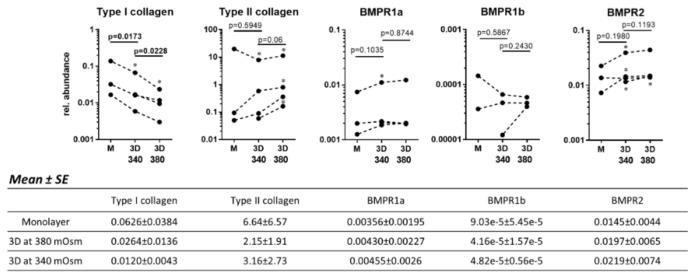
Human OA chondrocytes were cultured in a monolayer (M) for one week followed by three weeks in alginate (3D) at 340 or 380 mOsm. The gene expression of type I and type II collagen as well as of BMPR1a, b and BMPR2 was evaluated by RT-qPCR after the monolayer culture and after the alginate culture for each of the donors. Data on the graphs represent the mean of technical replicates (*n* = 3–6). Statistical analysis was performed for each donor separately (*means *p* < 0.05) and for all donors together with the *p* values being shown on the graphs (*p* values are bold when *p* ≤ 0.05). The means and standard errors of the mean (SE) for all donors together are tabulated below the graphs.

In contrast to the 3D culture, no effect of GDF-5 could be observed on the cell proliferation and GAG release with human OA chondrocytes cultured in a monolayer at 340 mOsm (not shown). To investigate this phenomenon, human OA chondrocytes were first cultured for one week in a monolayer and subsequently for three weeks in alginate at 340 or 380 mOsm. The expression of type I and II collagen as well as the expression of the GDF-5 receptors, BMPR2, BMPR1a and BMPR1b, were evaluated at the end of the monolayer and the 3D culture (Figure 5). 

As expected, type I collagen decreased (all donors together *p* = 0.0173) in all donors, and type II collagen expression increased in two of the three donors (not significant) when changing from monolayer to 3D culture at 340 mOsm. These effects were further enhanced at 380 mOsm. Indeed, in 3D culture, type I collagen expression was decreased at 380 mOsm compared to 340 mOsm (*p* = 0.0228) and type II collagen expression was increased in three of the four donors.

When comparing the expression of BMPRs in a monolayer versus 3D culture at 340 mOsm, the expressions of BMPR1a (significant in one donor) and BMPR2 (significant in two donors) were increased; however, the increase was not significant when considering all donors together. The expression of BMPR1b was low in comparison to BMPR1a and BMPR2, sometimes not detectable and showed discordant variations among donors when changing from monolayer to 3D culture. Increasing the osmolarity from 340 to 380 mOsm in 3D cultures had no clear effect on BMPR1a and 1b expression but there was a slight trend to an increase of BMPR2 expression (significant in one donor). However, in the monolayer, it was demonstrated that increasing osmolarity increases BMPR expression (Supplementary Figure S3).

It can be concluded that 3D culture and increasing medium osmolarity positively influences the chondrocyte phenotype and that 3D culture increases BMPR expression compared to monolayer culture. This increased BMPR expression might result in an elevated response to GDF-5.

### 2.6. The Presence of Inflammatory Cytokines Influences the BMPRs Expression of Human OA Chondrocytes 

**Figure 6:**
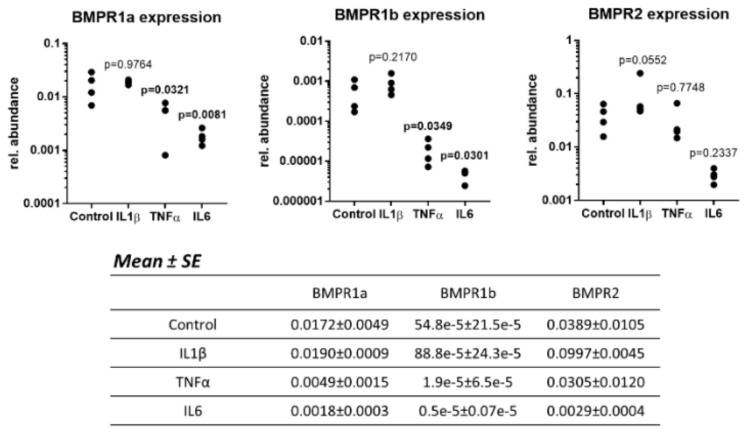
Human OA chondrocytes were cultured in a monolayer for two days in the presence of 10 ng/mL IL1*β*, 10 ng/mL TNFα or 100 ng/mL IL6 at 380 mOsm. Afterwards, the gene expression of BMPR1a, b and BMPR2 was evaluated by RT-qPCR. Data on the graphs represent the mean of technical replicates (*n* = 3–6). Statistical analysis was performed on biological replicates (*n* = 4 donors). *p* values are shown on the graphs and are bold when *p* ≤ 0.05, the means and standard errors (SE) of the mean are tabulated below the graphs.

It was previously shown that human OA chondrocytes produce cytokines and that cytokine levels were higher in monolayer than 3D culture. Therefore, we also aimed to evaluate the impact of inflammatory cytokines on BMPR expression. Human OA chondrocytes were cultured in a monolayer in the presence of IL1*β*, TNF*α* or IL6, or left untreated, and the expression of BMPR1a, 1b and 2 was measured (Figure 6). IL1*β* had no effect on the expression of BMPRs compared to unstimulated cells. On the contrary, TNFα and IL6 significantly down-regulated the expression of BMPR1a (*p* = 0.0321 and *p* = 0.0081, respectively) and BMPR1b (*p* = 0.0349 and *p* = 0.0301, respectively) compared to control cells, whereas they had no significant effect on BMPR2 expression. 

These results show that inflammatory cytokines decrease BMPR expression possibly resulting in a decreased responsiveness of the cells to GDF-5. 

## 3. Discussion

During the progression of OA, chondrocytes experience drastic modifications of their environment and their phenotype. OA chondrocytes produce proteases, which degrade the ECM and release cytokines that sustain the inflammation in the joint [2]. In addition, OA chondrocytes have a low responsiveness to growth factors [3,4,5]. One of the environmental parameters that is altered with the progression of the disease is osmolarity [7,8,9]. It was already known that osmolarities corresponding to healthy cartilage stabilizes the chondrocyte phenotype and ECM production [10,11,12,13], but its effect on the catabolic activity of chondrocytes or their inflammatory status was unknown. In this study, we aimed to further characterize the effect of osmolarity on the ‘OA’ characteristics of chondrocytes and their responsiveness to the growth factor GDF-5. 

### 3.1. Increasing the Osmolarity Stabilizes the Chondrocyte Phenotype, Stimulates Matrix Production and Reduces the Inflammatory Status of Human OA Chondrocytes

Human OA chondrocytes were first cultured in a monolayer at osmolarities ranging from 340 to 460 mOsm. It could be demonstrated that increasing the osmolarity increased GAG accumulation, aggrecan and type II collagen expression while decreasing type I collagen expression. This is in accordance with previous studies from Van der Windt et al. (2010, 2012) [12,20] who compared human OA chondrocytes cultured at 280 mOsm or at 380 mOsm. However, in the present study we could demonstrate that increasing osmolarity beyond 380 mOsm further amplifies the chondrocyte response. In addition, it was observed that ADAMTS5 and MMP13 expression, as well as TNF*α* and IL6 release, were decreased with increasing osmolarities. This indicates that higher osmolarities can decrease the catabolic and inflammatory status of human OA chondrocytes. To the best of our knowledge, this is the first time that this effect of the osmolarity on OA chondrocytes is described. 

Van der Windt et al. 2010 [12] demonstrated that at 480 and 580 mOsm, cell morphology and cell proliferation were severely affected. Similarly, we found that at 420 and 480 mOsm, cell proliferation was significantly reduced.

Based on these results it was decided to further evaluate the effect of osmolarity in 3D culture but at 380 mOsm only. It could be confirmed that at 380 mOsm, aggrecan expression increased and type I collagen expression decreased. In addition, in all four donors, type II collagen expression increased (significant in two donors out of four) and IL6 concentration decreased (significant in all four donors). On the contrary, no effect could be observed on ADAMTS5 and MMP13 expression, or on TNF*β* or IL1*α* release. In summary, in 3D culture, some but not all effects of osmolarity observed in a monolayer could be recapitulated.

### 3.2. The Combination of 3D Culture and 380 mOsm Permits a Robust Response of Human OA Chondrocytes to GDF-5

GDF-5 is known to have anabolic activities in healthy human chondrocytes [16,17,18,19] but leads to a discordant response in human OA chondrocytes [5]. Similarly, we could not observe any effect of GDF-5 in a monolayer of human OA chondrocytes (not shown). In 3D culture however, an increased proliferation and GAG accumulation was observed in the presence of GDF-5 (300 ng/mL) both at 340 and 380 mOsm but with a higher significance at 380 mOsm indicating a more robust response. The responsiveness of OA chondrocytes to GDF-5 in 3D in comparison to the monolayer culture might be explained by a better phenotype maintenance, lower cytokine concentrations and increased BMPR expression in 3D. The two last effects might be linked, as we demonstrated that IL6 and TNF*α* decrease BMPR expression. The reason for an increased responsiveness to GDF-5 at 380 mOsm compared to 340 mOsm in 3D culture is less evident but we observed a trend for an increased BMPR2 expression at 380 mOsm in monolayer and in 3D.

Based on these results, we suggest that the use of 3D cell culture with a slightly higher osmolarity (380 mOsm) in comparison to usual culture medium (340 mOsm) can facilitate investigation with human OA chondrocytes without compromising the validity of the results. With this culture system, the OA phenotype of the cell is dampened, and the phenotype might correspond to mid-stage instead of late-stage OA chondrocytes. Yet, mid-stage OA corresponds better to the intended treatment population for structure-modifying interventions (usually patients with Kellgren and Lawrence grade one to three are selected for treatment with structure-modifying drugs [21]).

### 3.3. Osmolarity is an Integral Part of the OA Pathobiology

Our results show that osmolarity influences matrix, protease and cytokine production by chondrocytes and modulates their response to GDF-5. It strongly indicates that decreased osmolarity in OA cartilage can be one of the factors sustaining the progression of the disease. Clinically it might imply that increasing the osmolarity in the cartilage with the injection of a hyper-osmotic solution intra-articularly could improve OA. However, because osmolarity in cartilage is governed by the fixed charge density from the GAG [8] and the Donnan equilibrium between the tissue and its bathing solution, such an injection is not likely to have any effect. Only matrix regeneration can durably increase the fixed charge density and osmolarity in cartilage.

Finally, to confirm the role of osmolarity in the OA pathology it would have been interesting to also evaluate if reducing the osmolarity below 340 mOsm increases the production of cytokines and proteases in healthy or OA chondrocytes.

In summary, it was demonstrated that osmolarity is a critical parameter of the OA pathobiology. Modulating osmolarity in cartilage in vivo might be very challenging and is not likely to become a possible therapeutic approach. However, modulating osmolarity in vitro might further reveal new aspects of the OA chondrocyte biology.

## 4. Materials and Methods

### 4.1. Medium Preparation and Osmolarity Measurement

The culture medium was DMEM high glucose (Gibco from Thermofisher scientific, Waltham, MA, USA) supplemented with 10% fetal bovine serum (Biochrom, Berlin, Germany), 50 µg/mL ascorbic acid (Millipore Sigma, Saint Louis, MI, USA) and 400 µM proline (Millipore Sigma). The osmolarity of this medium was about 340 (339 ± 4.9 mOsm after verification) mOsm and was adjusted to 380 (377 ± 4.7 mOsm), 420 (415 ± 6.0 mOsm) or 460 (462 ± 4.4 mOsm) mOsm using 250 mg/mL sterile NaCl solution (Merck KGaA, Darmstadt, Germany). The osmolarity was verified with a cryoscopic osmometer (Osmomat 030-3P from Gonotec, Berlin, Germany).

### 4.2. Human OA Chondrocyte Isolation

The isolation of human OA chondrocytes was performed from the cartilage of patients who underwent a total knee or hip replacement surgery. Human material was provided by the clinic for orthopedics, traumatology and sports medicine in Elisabethenstift Darmstadt with full, ethical, written consent (ethical approval No. FF24/2015, approved by the ethic committee of the State Chamber of Physicians from Hessen, in March 2015). In our hands, no difference could be observed between the behavior of hip or knee chondrocytes and cells originating from both joints used for this study. The cartilage originated at 68.75% from female donors and 31.25% from male donors. The average age of the donors was 70.3 ± 9 years old. The entire cartilage tissue was harvested and digested to isolate the chondrocytes. Briefly, the cartilage was digested sequentially with 0.25% *w*/*v* collagenase (Serva GmbH, Heidelberg, Germany) in HAM’s F12 (Gibco from Thermofisher scientific, Waltham, MA, USA) for 30 min at room temperature and 0.1% *w*/*v* collagenase in HAM’s F12 + 1% penicillin/streptomycin (PAN Biotech, Aidenbach, Germany) overnight at 37 °C. The resulting cell suspension was filtered through 100 µm, then 40 µm cell strainers (Becton Dickinson GmbH, Heidelberg, Germany), washed several times by centrifugation and resuspended in culture medium. After each isolation, cells were sampled for gene expression analysis and are described as freshly isolated chondrocytes (FIC).

### 4.3. Monolayer Cultures at 340, 380, 420 or 460 mOsm

Human OA chondrocytes were plated directly after isolation in culture medium at 340 mOsm (= unchanged osmolarity) or in culture medium with an osmolarity adjusted to 380, 420 or 460 mOsm in PRIMARIA™ 24 well plates (Corning, New York, NY, USA) at 240,000 cells/well. The media were renewed after three or four days and cells were cultured for six days. A total of five donors were used and for each donor a minimum of six cell cultures were realized for each condition; three were used for determination of the cell concentration (resulting in a minimum of *n* = 3 technical replicates per donors) and the three others for gene expression analysis (resulting in *n* = 3 technical replicates per donors or *n* = 2 when RNA isolation was not successful in one of the samples). The GAG (*n* = 3–6 per donor) and cytokine concentrations (*n* = 3–6 per donor) were analyzed in the medium. 

### 4.4. Monolayer Cultures in the Presence of Cytokines

Isolated human OA chondrocytes were first cultured for seven days in a monolayer. Therefore, 8–10 × 10^6^ cells were seeded into PRIMARIA™ T-75 flasks (Corning) in the culture medium with 1% penicillin/streptomycin (PAN biotech). Afterwards, the cells were harvested and seeded at a concentration of 0.4 × 10^6^ cells/mL into 24-well plates (Falcon from Thermofisher scientific, Waltham, MA, USA) containing the culture medium adjusted to 380 mOsm. After one day of culture, the medium was changed for the same medium but devoid of serum and containing 10 ng/mL IL1*β* (Millipore Sigma, Saint Louis, MI, USA, Cat. N° I9401) and TNF*α* (R&D Systems, Minneapolis, MI, USA, Cat. N° 210 TA/CF) or 100 ng/mL IL6 (R&D Systems, Minneapolis, MI, USA, Cat. N° 206-IL/CF). A control culture without cytokine was also realized. After two days, cell samples were analyzed for gene expression (real-time PCR, *n* = 3–4 per donor) analysis. A total of four donors were used.

### 4.5. Alginate Bead Cultures at 340 and 380 mOsm

Cells were first cultured for one week in a monolayer as described above before being encapsulated in alginate. To do so, cells were resuspended in alginate gel (0.2 M HEPES from AppliChem GmbH, Darmstadt, Germany, 1.5 M NaCl from Merck KGaA, Darmstadt, Germany and 1.25% alginate from Millipore Sigma, Saint Louis, MI, USA, autoclaved and adjusted to pH 7.4) at 2 × 10^6^ cells/mL and poured drop by drop through a 22-g needle attached to a 10 mL syringe into the polymerization solution (120 mM CaCl_2_ from Merck and 10 mM HEPES). After 15 min under agitation, the drops or alginate beads were completely polymerized and washed three times with 150 mM NaCl solution. The beads were divided and placed into four different petri dishes containing 20 mL of the culture medium at 340 mOsm or adjusted to 380 mOsm. The beads were cultured for seven days at 37 °C. Afterwards, the beads were carefully transferred in 24 well ultra-low attachment plates (VWR, Radnor, PA, USA, 5 beads/well) containing 1 mL of the respective media. For growth factor experiments, these media were supplemented with 300 ng/mL GDF-5 (kindly provided by Biopharm GmbH, Eppelheim, Germany as a 3.94 mg/mL solution in 10 mM HCl) or 12.5 µM HCl (Merck KGaA, used as control). The total culture period was 14 days and the media were renewed twice a week. At the end of the culture period, medium samples were analyzed for their cytokine concentration (*n* = 3–4 per donor). The alginate beads were dissolved. To do so, beads were washed with PBS (Gibco from Thermofisher scientific, Waltham, MI, USA) and dissolved in 460 µL dissolution solution (55 mM Na-citrate from Merck and 150 mM NaCl at pH 8) together with 40 µL 2.5% collagenase (diluted in DMEM high glucose containing 0.2 mM CaCl_2_) for one hour at 37 °C. Afterwards, 500 µL PBS or DMEM high glucose was added and the solution centrifuged. The supernatant was analyzed for its GAG content (*n* = 3–8) and the cells were counted (*n* = 3–4) or used for gene expression analysis (*n* = 3–6). A total of four donors were used.

### 4.6. Cell Concentration and Viability

The cell concentration and the cell viability were measured with a Vi-Cell XR analyzer (Beckmann-Coulter, Brea, CA, USA). The trypan blue dye exclusion method was used to differentiate living and dead cells.

### 4.7. Glycosaminoglycan Analysis

For GAG quantification, the dimethylmethylene blue assay was used. The absorbance of the samples was measured at 540/595 nm using a Paradigm MTP Reader (Beckmann-Coulter) and compared to that of chondroitin sulfate standards (Millipore Sigma).

### 4.8. Cytokine Analysis

For cytokine measurements, two different sandwich multi-spot immunoassays (Mesoscale Discovery, Rockville, MD, USA) were performed according to the manufacturer’s protocol. The 4-spot proinflammatory tissue culture array was used for monolayer samples, whereas the 10-spot proinflammatory panel was used for alginate bead samples.

### 4.9. Reverse-Transcription Quantitative PCR (RT-qPCR)

Cell lysis was performed in RLT buffer (RNeasy Mini Kit, Qiagen, Hilden, Germany). Cells obtained from alginate beads were additionally treated with proteinase K (RNeasy Mini Kit, Qiagen). Ribonucleic acid (RNA) was extracted using the RNeasy Mini Kit (Qiagen). Afterwards, RNA concentration and integrity were determined by a capillary gel electrophoresis using an Agilent 600 Nano Chip with an Agilent 2100 Bioanalyzer. The isolated RNA was reverse transcribed by using the SuperScript III First-Stranded Synthesis Supermix Kit (Invitrogen from Thermofisher scientific; Waltham, MA, USA). Quantitative PCR (qPCR) was realized with the SYBR-Green Jumpstart Taq Ready Mix (Sigma) by using 250 nM of the forward and reverse primer (from Eurofins MWG Operon, Ebersberg, Germany, see supplementary Table S1). The PCR reaction was run in the thermocycler Mx3000P (Agilent, Santa Clara, CA, USA) and each sample was analyzed in duplicates against EF1α (housekeeping gene). Afterwards the relative abundance of the target genes was calculated by using the following formula: relative abundance = 2^(CT^_HKG_^-CT^_GOI_^)^; with CT = cycle threshold, HKG = housekeeping gene and GOI = gene of interest.

### 4.10. Statistical Analysis

Statistical analysis was performed on the results obtained for each donor separately (technical replicates) or all donors together (biological replicates) with the software GraphPad Prism v7.00 (GraphPad Software, San Diego, CA, USA). A repeated measurement one-way ANOVA was performed with a correction for multiple comparison (Dunnett test) to compare several groups or a t-test to compare two groups.

## Supplementary Materials

Supplementary materials can be found at https://www.mdpi.com/1422-0067/21/2/531/s1.

## Abbreviations

ADAMTS5	A Disintegrin And Metalloproteinase with ThromboSpondin motifs 5
ANOVA	Analysis Of Variance
BMP	Bone Morphogenic Protein
ECM	Extra-cellular Matrix
FIC	Freshly Isolated Chondrocytes
GAG	GlycosAminoGlycan
GDF-5	Growth and Differentiation Factor-5
HEPES	4-(2-hydroxyethyl)-1-piperazineethanesulfonic acid
IGF1	Insulin-like Growth Factor 1
IL1*β*	InterLeukin 1*β*
IL6	InterLeukin 6
MMP13	Matrix Metallopeptidase 13
OA	Osteoarthritis
PCR	Polymerase Chain Reaction
TGF*β*	Transforming Growth Factor *β*
TNF*α*	Tumor Necrosis Factor *α*

## References

[1] Alcaraz M.J., Guillén M.I., Ferrándiz M.L. (2019). Emerging therapeutic agents in osteoarthritis. Biochem. Pharmacol..

[2] Aigner T., Söder S., Gebhard P.M., McAlinden A., Haag J. (2007). Mechanisms of Disease: Role of chondrocytes in the pathogenesis of osteoarthritis - Structure, chaos and senescence. Nat. Clin. Pract. Rheumatol..

[3] Loeser R.F., Shanker G. (2000). Autocrine stimulation by insulin-like growth factor 1 and insulin-like growth factor 2 mediates chondrocyte survival in vitro. Arthritis Rheum..

[4] Blaney Davidson E., Scharstuhl A., Vitters E., van der Kraan P., van den Berg W., Moos V., Fickert S., Muller B., Weber U., Sieper J. (2005). Reduced transforming growth factor-beta signaling in cartilage of old mice: Role in impaired repair capacity. Arthritis Res. Ther..

[5] Ratnayake M., Plöger F., Santibanez-Koref M., Loughlin J. (2014). Human chondrocytes respond discordantly to the protein encoded by the osteoarthritis susceptibility gene GDF5. PLoS One.

[6] Grushko G., Schneiderman R., Maroudas A. (1989). Some biochemical and biophysical parameters for the study of the pathogenesis of osteoarthritis: A comparison between the processes of ageing and degeneration in human hip cartilage. Connect. Tissue Res..

[7] Shanfield S., Campbell P., Baumgarten M., Bloebaum R., Sarmiento A. (1988). Synovial fluid osmolality in osteoarthritis and rheumatoid arthritis. Clin. Orthop. Relat. Res..

[8] Maroudas A., Ziv I., Weisman N., Venn M. (1985). Studies of hydration and swelling pressure in normal and osteoarthritic cartilage. Biorheology.

[9] Urban J.P.G. (1994). The chondrocyte: A cell under pressure. Rheumatology.

[10] Negoro K., Kobayashi S., Takeno K., Uchida K., Baba H. (2008). Effect of osmolarity on glycosaminoglycan production and cell metabolism of articular chondrocyte under three-dimensional culture system. Clin. Exp. Rheumatol..

[11] Urban J.P.G., Hall A.C., Gehl K.A. (1993). Regulation of matrix synthesis rates by the ionic and osmotic environment of articular chondrocytes. J. Cell. Physiol..

[12] Van Der Windt A.E., Haak E., Das R.H.J., Kops N., Welting T.J.M., Caron M.M.J., Van Til N.P., Verhaar J.A.N., Weinans H., Jahr H. (2010). Physiological tonicity improves human chondrogenic marker expression through nuclear factor of activated T-cells 5 in vitro. Arthritis Res. Ther..

[13] Xu X., Urban J.P.G., Tirlapur U.K., Cui Z. (2010). Osmolarity effects on bovine articular chondrocytes during three-dimensional culture in alginate beads. Osteoarthr. Cartil..

[14] Katagiri T., Watabe T. (2016). Bone Morphogenetic Proteins. Cold Spring Harb. Perspect. Biol..

[15] Parrish W.R., Byers B.A., Su D., Geesin J., Herzberg U., Wadsworth S., Bendele A., Story B. (2017). Intra-articular therapy with recombinant human GDF5 arrests disease progression and stimulates cartilage repair in the rat medial meniscus transection (MMT) model of osteoarthritis. Osteoarthr. Cartil..

[16] Appel B., Baumer J., Eyrich D., Sarhan H., Toso S., Englert C., Skodacek D., Ratzinger S., Grässel S., Goepferich A. (2009). Synergistic effects of growth and differentiation factor-5 (GDF-5) and insulin on expanded chondrocytes in a 3-D environment. Osteoarthr. Cartil..

[17] Chubinskaya S., Segalite D., Pikovsky D., Hakimiyan A.A., Rueger D.C. (2008). Effects induced by BMPS in cultures of human articular chondrocytes: Comparative studies. Growth Factors.

[18] Enochson L., Stenberg J., Brittberg M., Lindahl A. (2014). GDF5 reduces MMP13 expression in human chondrocytes via DKK1 mediated canonical Wnt signaling inhibition. Osteoarthr. Cartil..

[19] Murphy M.K., Huey D.J., Hu J.C., Athanasiou K.A. (2015). TGF-β1, GDF-5, and BMP-2 stimulation induces chondrogenesis in expanded human articular chondrocytes and marrow-derived stromal cells. Stem Cells.

[20] Van Der Windt A.E., Haak E., Kops N., Verhaar J.A.N., Weinans H., Jahr H. (2012). Inhibiting calcineurin activity under physiologic tonicity elevates anabolic but suppresses catabolic chondrocyte markers. Arthritis Rheum..

[21] Mc Alindon T.E., Driban J.B., Henrotin Y., Hunter D.J., Jiang G.L., Skou S.T., Wang S., Schnitzer T. (2015). OARSI Clinical Trials Recommendations: Design, conduct, and reporting of clinical trials for knee osteoarthritis. Osteoarthr. Cartil..

