# Supplementary material for: Increasing the Medium Osmolarity Reduces the Inflammatory Status of Human OA Chondrocytes and Increases Their Responsiveness to GDF-5
Source: Int J Mol Sci. 2020 Jan 14;21(2):531. doi: 10.3390/ijms21020531 (PMC7014320; doi:10.3390/ijms21020531)
Supplement: Supplementary file 1 [file ijms-21-00531-s001.pdf]

**Table S1**. PCR primers.

| | |
|---|---|
| Human EF1α | Forward: 5'- CCTTGTGGAAATTTGAGACC -3'<br>Reverse: 5'- CCATTTTGTTAACACCGACA -3' |
| Human ADAMTS5 | Forward: 5'- TCAAAGCCAAAGACCAGACT -3'<br>Reverse: 5'- ATTTCCTTCGTGGCAGAGTA -3' |
| Human Aggrecan | Forward: 5'- GAAAGGCATCGTGTTCCATT -3'<br>Reverse: 5'- ACGTCCTCACACCAGGAAAC -3' |
| Human BMPR1a | Forward: 5'- CAGGTTCCTGGACTCAGCTC -3'<br>Reverse: 5'- CTTTCCTTGGGTGCCATAAA -3' |
| Human BMPR1b | Forward: 5'- AAAGGTCGCTATGGGGAAGT -3'<br>Reverse: 5'- GCAGCAATGAAACCCAAAAT -3' |
| Human BMPR2 | Forward: 5'- GCTAAAATTTGGCAGCAAGC -3'<br>Reverse: 5'- CTTGGGCCCTATGTGTCACT -3' |
| Human Collagen 1 | Forward: 5'- AAAGGATCTCCTGGTGAAGC -3'<br>Reverse: 5'- CACCTTTAGGTCCAGGGAAT -3' |
| Human Collagen 2 | Forward: 5'- CCTGAGTGGAAGAGTGGAGA -3'<br>Reverse: 5'- TCCATAGCTGAAATGGAAGC -3' |
| Human MMP13 | Forward: 5'- CCAACCCTAAACATCCAAAAAC -3'<br>Reverse: 5'- AAAAACAGCTCCGCATCAAC -3' |

**Figure S1.** Human OA chondrocytes from five donors were cultured six days in monolayer at 340, 380, 420 and 480 mOsm. The cell viability was evaluated. Data on the graph represent the mean +/- standard error of the mean of technical replicates (n=3-6). Statistical analysis was performed for each donor separately (* means significantly different from 340 mOsm with $p<0.05$) and for all donors together, with the means, standard errors of the mean (SE) and P values for the comparison to 340 mOsm shown in the table.

**Cell viability**

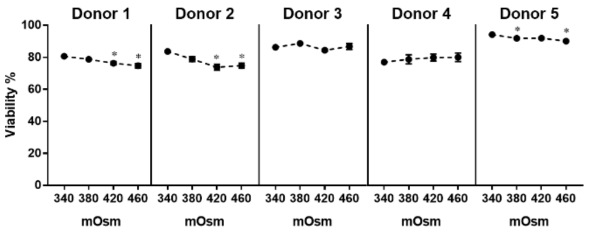


***Statistics for all donors together***

| | Mean ± SE | P-value |
|---|---|---|
| 380 mOsm | 84.3±2.9 | |
| 380 mOsm | 83.36±2.9 | 0.8555 |
| 420 mOsm | 81.3±3.2 | 0.1531 |
| 460 mOsm | 81.3±3.1 | 0.1528 |

**Figure S2.** Human OA chondrocytes from two donors were cultured six days in monolayer at 340, 380, 420 and 480 mOsm and (osmolarity was adjusted with sucrose). The GAG release as well as IL1β, IL6 and TNFα concentrations were measured in the medium. Cell samples were used to evaluate the cell concentration, cell viability and gene expression of aggrecan, type II and I collagen, ADAMTS5, MMP13 and BMPR1a, 1b and 2 by RT-qPCR. Data represent the mean of technical replicates (n=3-6). * means significantly different from 340 mOsm with p<0.05,

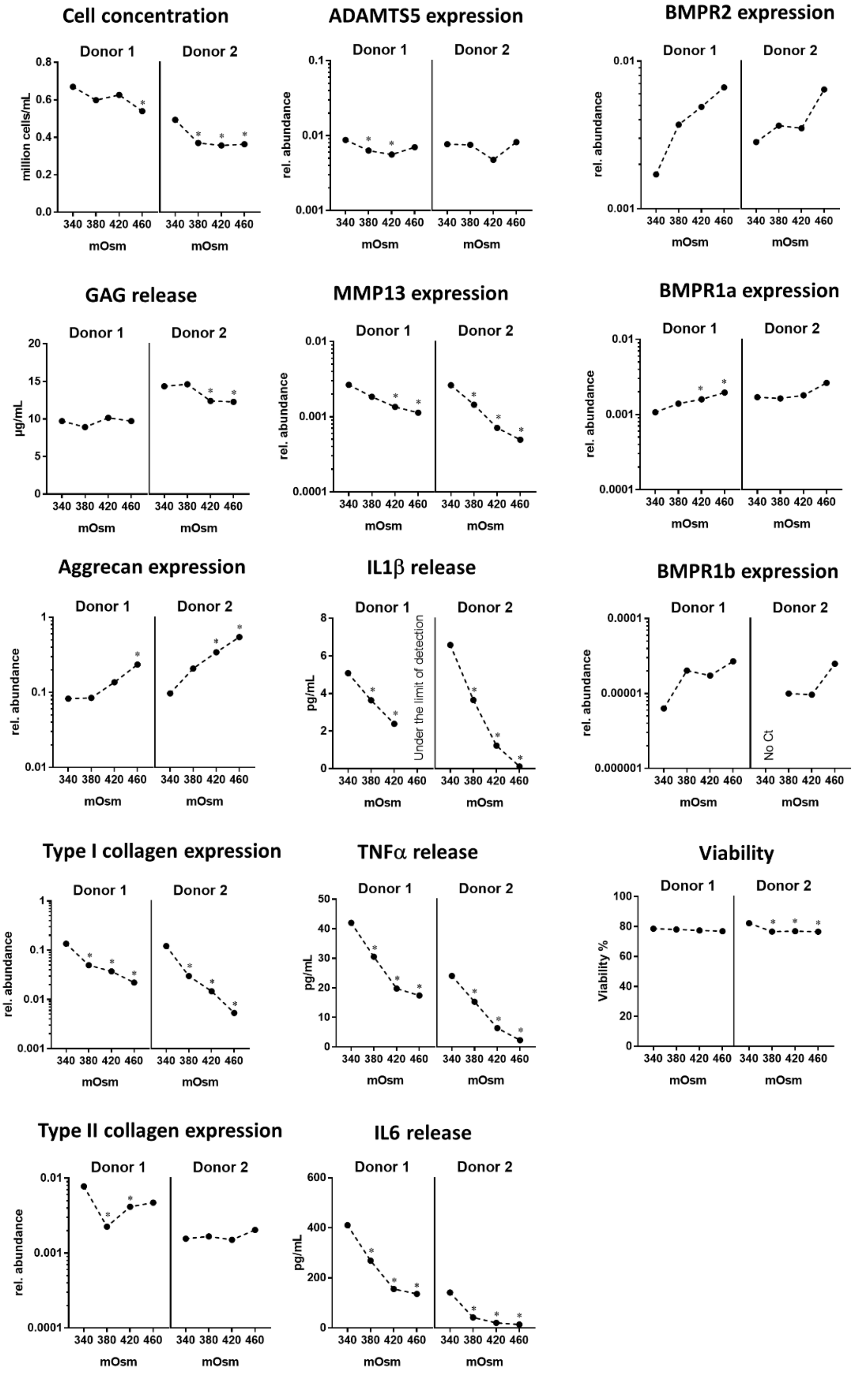

**Figure S3.** Human OA chondrocytes from five donors were cultured six days in monolayer at 340, 380, 420 and 480 mOsm. The gene expression of BMPR1a, 1b and 2 was evaluated by RT-qPCR. Data on the graphs represent the mean of technical replicates (n=3-6). The level of expression in FIC is shown with a dotted line. Statistical analysis was performed for each donor separately (*means statistically different from 340 mOsm with p<0.05) and for all donors together, with the means, standard errors of the mean (SE) and P values for the comparison to 340 mOsm shown in the table.

**BMPR2**

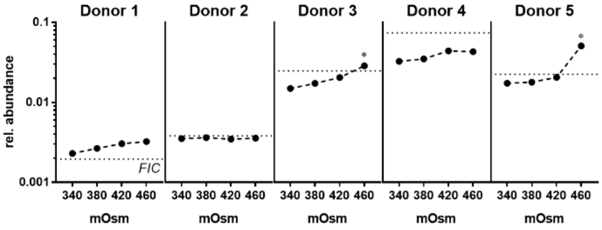


***Statistics for all donors together***

| | Mean ± SE | P-value |
|---|---|---|
| 380 mOsm | 0.0141±0.0054 | |
| 380 mOsm | 0.0153±0.0059 | 0.9873 |
| 420 mOsm | 0.0183±0.0075 | 0.6640 |
| 460 mOsm | 0.0258±0.0098 | 0.0506 |

**BMPR1a**

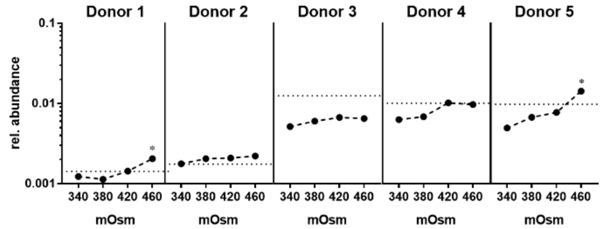


| | Mean ± SE | P-value |
|---|---|---|
| 380 mOsm | 0.00388±0.00100 | |
| 380 mOsm | 0.00455±0.00123 | 0.8731 |
| 420 mOsm | 0.00563±0.0009 | 0.2994 |
| 460 mOsm | 0.00694±0.0023 | **0.0414** |

**BMPR1b**

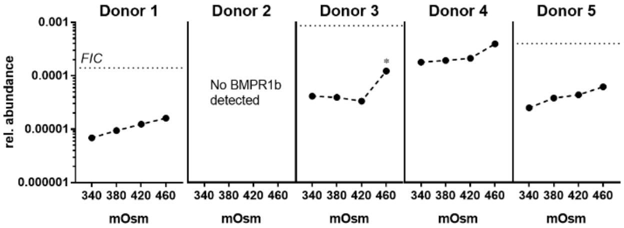


| | Mean ± SE | P-value |
|---|---|---|
| 380 mOsm | 6.34e-5±3.95e-5 | |
| 380 mOsm | 7.03e-5±4.18e-5 | 0.9924 |
| 420 mOsm | 7.55e-5±4.60e-5 | 0.9615 |
| 460 mOsm | 15e-5±8.53e-5 | 0.0557 |
